# Lipid profile changes in erythrocyte membranes of women with diagnosed GDM

**DOI:** 10.1371/journal.pone.0203799

**Published:** 2018-09-14

**Authors:** Malgorzata Bukowiecka-Matusiak, Izabela Burzynska-Pedziwiatr, Anna Sansone, Beata Malachowska, Monika Zurawska-Klis, Carla Ferreri, Chryssostomos Chatgilialoglu, Tomasz Ochedalski, Katarzyna Cypryk, Lucyna Alicja Wozniak

**Affiliations:** 1 Medical University of Lodz, Department of Structural Biology, Lodz, Poland; 2 Consiglio Nazionale delle Ricerche, Institute for the Organic Synthesis and Photoreactivity, Bologna, Italy; 3 Medical University of Lodz, Department of Biostatistics and Translational Medicine, Lodz, Poland; 4 Medical University of Lodz, Department of Nursing and Obstetrics, Department of Clinic Nursing, Department of Diabetology and Metabolic Diseases Lodz, Poland; 5 Medical University of Lodz, Department of Comparative Endocrinology, Lodz, Poland; University of Rochester, UNITED STATES

## Abstract

Gestational diabetes mellitus (GDM) is a glucose intolerance that begins or is first recognized during pregnancy. It is currently a growing health problem worldwide affecting from 1% to 14% of all pregnant women depending on racial and ethnic group as well as the diagnostic and screening criteria. Our preliminary study aimed at investigating the erythrocyte membrane fatty acid profiles of pregnant women, in particular with diagnosed with gestational diabetes mellitus (GDM), and with normal glucose tolerant (NGT) pregnant women as a control group. The study group comprised 43 pregnant women, 32 of whom were diagnosed with GDM according to the WHO criteria, and 11 with normal glucose tolerance. The erythrocyte membrane phospholipids were obtained according to the Folch extraction procedure. Fatty acids (FA) were analyzed by gas chromatography (GC) as the corresponding fatty acid methyl esters (FAME). A cluster of 14 fatty acids identified contained >98% of the recognized peaks in the GC analysis. The analysis of fatty acids from erythrocytes revealed important differences between GDM and NGT women in the third trimester, and the results were correlated with biochemical data. Among the 14 measured FA representing the membrane lipidomic profile, the levels of three saturated FA (myristic, palmitic, stearic acids) tended to decrease in GDM patients, with the percentage content of stearic acid significantly changed. The relative content of monounsaturated fatty acids (MUFA) tended to increase, in particular the oleic acid and vaccenic acid contents were significantly increased in erythrocyte membranes of the GDM group in comparison with the NGT group. The GDM group demonstrated higher sapienic acid levels (+29%) but this change was not statistically significant. This study revealed association between an impaired *cis*-vaccenic acid concentration in erythrocytes membrane and GDM development. No significant changes of polyunsaturated fatty acids (PUFA) were observed in GDM and NGT erythrocytes. We postulate, basing on the differences between the GDM and NGT lipidomic profiles, that stearic and *cis-*vaccenic acids can be considered as dual biomarkers of specific SFA-MUFA conversion pathway, involving the coupling of delta-9 desaturase and elongase enzymes. Our results indicate that the SFA-MUFA families may be involved in the pathophysiology of metabolic diseases such as GDM, but the further studies are needed to confirm our hypothesis. In conclusion, the erythrocyte membranes of GDM women undergo remodeling resulting in abnormal fatty acid profiles, which are reflection of the long-term status of organism and can have great impact on both the mother and her offspring.

## Introduction

Gestational diabetes mellitus (GDM) is defined as glucose intolerance that begins or is first recognized during pregnancy. Currently, GDM is the most frequent complication in pregnancy and has been attracting growing attention. Depending on the population, it affects from 1 to 14% of all pregnant women [1]. GDM increases the risk of numerous complications for both the mother (e.g. preeclampsia, preterm delivery, pregnancy hypertension) and her offspring (e.g. macrosomia and hypoglycemia) [2]. It has been reported that the incidence of GDM increases the risk of developing diabetes by about 9.6 times for patients with a previous history of GDM, with a cumulative risk of 25% within 15 years following diagnosis [3]. Women with GDM face an increased risk of cardiovascular diseases [4] and their offspring face a greater risk of developing obesity and abnormal glucose metabolism throughout their entire life [5]. Therefore, early diagnosis and treatment of GDM is crucial since the occurrence of hyperglycemia in pregnancy predisposes the developing fetus to poor metabolic health later in life [6].

For several years, our research activities have focused on molecular aspects of GDM [7, 8, 9] and high-throughput screening “omics” approaches, in particular transcriptomics and metabolomics; these provide a clearer insight into the pathogenesis of GDM and postpartum developing type 2 diabetes (T2DM) in women, thus allowing for the selection of candidate genes as early biomarkers. Moreover, in recent years, membrane fatty acid-based functional lipidomics has become a convenient and relevant molecular tool for examining the nutritional-metabolic status of patients compared to healthy controls, thus allowing lipidomic phenotypes to be identified in healthy and sick subjects [10, 11]. While the presence of reduced amounts of choline-containing lipids (phosphatidylcholines) circulating in umbilical cord blood is known to be predictive of type 1 diabetes developed later in infants [12], no study has yet examined the erythrocyte membrane fatty acid profile in GDM women [13]. On the other hand, dyslipidemia reported in women with T2DM is associated with significant changes in phosphatidylcholine lipid species in the circulating lipoproteins [14]. It is worth underlining that lipidomic profiles can be obtained from different body tissues and body fluids, and the analytical approach using mass spectrometry-based techniques is generating copious data processed by bioinformatics tools. In such sophisticated analyses, the saturated-monounsaturated pathways have been clearly evidenced in the insulin resistance connected to T2DM, also as elements from the diets [15, 16].

On the other hand, fatty acids when considered as constituents of cell membrane phospholipids, can be defined as a cluster with a restricted and specific number of molecules which are strictly connected with the multifaceted, biological roles of the membranes compartment itself. In cell membranes, the types and quantities of fatty acids are key factors in homeoviscous adaptation, which includes the modulation of biophysical, biochemical and signaling processes, thus implementing sensing mechanisms and stimuli transduction, and participating in overall epigenetic control pathways.

Membrane fatty acid-based functional lipidomics is a useful tool in molecular diagnostics that examines the levels of SFA and monounsaturated fatty acids (MUFA), either those synthesized endogenously or those obtained from the diet, as well as polyunsaturated fatty acids (PUFA) whose intake is essential for humans, with the assumption of precursors from the diet [11]. Since all fatty acids are vital structural components of membrane phospholipids, they are necessary for proper fetus development [17]. Fatty acids content in plasma correlates with the ongoing diet, whereas their membrane lipid content occurs as a result of membrane structure and remodeling as the effect of physiological and pathological changes towards the homeostatic balance [10].

In particular, MUFAs are biologically and pathophysiological significant molecules, especially for metabolic diseases. They are responsible for membrane fluidity, cell proliferation, lipid-mediated cytotoxicity, pathogenesis of obesity and cancer, programmed cell death and the unfolded protein response [18, 19, 20]. The main MUFAs are oleic, palmitoleic and vaccenic acids (VA). A recent study suggested an inverse association between the synthesis of VA in obese mice and the gluconeogenesis [21]

Moreover, a higher concentration of VA in red blood cells is connected with a reduced risk of T2DM, lower fasting glucose and better insulin sensitivity [22].

Taking into account that GDM is a metabolic disease that occurs quite suddenly in an otherwise healthy woman, early lipidomic evaluation could evidence the initial pathway of molecular imbalance, which is of particular significance when examining the membranes and their composition. The aim of the present study, therefore, is to investigate the erythrocyte membranes fatty acid profile in GDM versus pregnant women without carbohydrate disturbance, and by doing so, envisage the presence of a specific fatty acid pathway as a biomarker of the molecular transformations involved in the onset of GDM.

## Methods

### Study design

43 pregnant women were recruited into the reported study. Of these, 32 were diagnosed with GDM according to WHO—2013 criteria, according to which GDM should be diagnosed at any time in pregnancy if one or more of the following criteria are met: fasting plasma glucose 5.1–6.9 mmol/L (92–125 mg/dl); 1-hour plasma glucose ≥ 10.0 mmol/L (180 mg/dL) following a 75g oral glucose load; 2-hour plasma glucose 8.5–11.0 mmol/L (153–199 mg/dL) following a 75g oral glucose load [23].

(GDM group) and 11 had normal glucose tolerance (NGT group). Patients were recruited between 24^th^ and 28^th^ weeks of pregnancy after the OGTT test.

The study was conducted according to the guidelines of the Declaration of Helsinki and was approved by the Ethical Committee of the Medical University of Lodz (No. KB/268/15 from 17 February 2015). Informed consent was obtained from all participating subjects.

The inclusion criteria were as follows: Caucasian ethnic background, age 25–35 years, no GDM in previous pregnancy, no family history of diabetes in first-degree relatives, absence of concomitant diseases (chronic or acute infections), not taking insulin or other hypoglycemic medications.

Blood samples obtained from GDM and NGT women after a 12-hour overnight fasting were collected in Na_2_-EDTA (10 mL) vacutainers. The biochemical and clinical characteristics of GDM and NGT groups are given in Table 1.

**Table 1:** Biochemical and clinical parameters of the GDM and NGT groups.

	GDM*	NGT*	*p*
Age	31.0 (28–35)	29.0 (28–30)	0.1144
BMI [kg/m2]	23.7 (21.4–26.3)	20.9 (20.4–21.3)	0.0258
FPG [mg/dL]	85.0 (79.0–92.0)	77.5 (75.1–84.0)	0.0616
**OGTT 120’ [mg/dL]**	160.0 (153.0–172.0)	99.6 (88.8–100.9)	**<0.0001**
**CRP [mg/L]**	3.5 (2.3–8.3)	1.5 (1.1–2.5)	**0.0119**
Insulin [μU/mL]	10.7 (6.6–19.8)	13.9 (7–15.5)	0.7746
HOMA-IR	2.0 (1.5–3.8)	2.3 (1.3–3.2)	0.5899
HOMA-ß	203.3 (119.5–241.9)	287.3 (208.5–344.0)	0.0573
QUICKI	0.34 (0.032–0.37)	0.34 (0.31–0.36)	0.5899
**Total cholesterol [mg/dL]**	259.9 (233.3–283.4)	219.5 (191.5–242.0)	**0.0070**
HDL [mg/dL]	74.1 (57.0–86.0)	61.4 (53.9–67.1)	0.0802
LDL [mg/dL]	141.0 (116.0–173)	119.0 (105.0–139.0)	0.1567
**TG [mg/dL]**	215.9 (165.5–251.5)	157.6 (116.5–203.9)	**0.0398**

* Median with IQR

IQR- interquartile range

BMI–body mass index; FPG–fasting plasma glucose; OGTT–oral glucose tolerance test; CRP–C reactive protein; HOMA-IR—homeostasis model assessment of insulin resistance; HOMA- β–homeostasis model assessment of beta cells; QUICKI–quantitative insulin sensitivity check index; HDL—high-density lipoprotein; LDL—low-density lipoprotein; TG—triglycerides.

### Fatty acid analysis

The erythrocyte membrane phospholipids were isolated as described previously [20]. Fatty acids were converted to the corresponding fatty acid methyl esters (FAME) and analyzed by gas chromatography (GC). A cluster of 14 fatty acids was individuated that formed >98% of the recognized peaks in the GC analysis. The percentages were adjusted by the calibration factors obtained by the quantitative protocol run for each fatty acid type, using palmitic acid as an external standard (at 2.5 mM concentration; peak area = 468.2) with calibration factor = 1. Each fatty acid area was obtained by the GC and, together with the calibration factor, were used in the following equation:

$$\left(\frac{GC\;area\times factor\times molarity\;of\;palmitic\;acid}{GC\;area\;of\;palmitic\;acid}\right)\times molar\;mass\;of\;the\;appropriate\;acid$$

The results are obtained in relative percentages in the cluster of 14 fatty acids and presented in Table 2.

**Table 2:** Erythrocyte membrane lipidomic profile using 14 fatty acids as a representative cluster.

Fatty acid^$^	GDM*	NGT*	Δ(%) **	*p*	FDR
	**Saturated fatty acids (SFA)**				
Myristic (C14:0)	0.16 (0.13–0.21)	0.17 (0.14–0.21)	-6%	0.5735	0.7299
Palmitic (C16:0)	16.87 (16.34–17.39)	17.35 (16.08–17.47)	-3%	0.4496	0.7254
**Stearic** (C18:0)	11.75 (10.99–12.31)	12.13 (11.94–12.87)	**-3%**	**0.0487**	**0.3408**
	**Monounsaturated fatty acids (MUFA)**				
Sapienic (C16:1)	0.21 (0.11–0.34)	0.15 (0.13–0.17)	+29%	0.1041	0.4573
Palmitoleic (C16:1(9*cis*))	0.24 (0.19–0.48)	0.23 (0.12–0.48)	+4%	0.4663	0.7254
Oleic (C18:1 (9cis))	18.25 (17.72–19.22)	18.65 (17.78–19.47)	-2%	0.6507	0.7591
**Vaccenic (C18:1 (11*cis*))**	1.28 (1.15–1.49)	1.10 (0.96–1.18)	**+14%**	**0.0071**	**0.1001**
	**Polyunsaturated fatty acids (PUFA)**				
Linoleic (C18:2 (9,12))	13.04 (11.79–14.03)	13.47 (12.80–14.78)	-3%	0.2521	0.5769
Alpha-linolenic (C18:2 (9,12,15))	0.49 (0.40–0.61)	0.41 (0.35–0.47)	+16%	0.1307	0.4573
Gamma-linolenic (C18:3 (6,9,12))	2.51 (2.27–2.92)	2.13 (1.94–2.96)	+15%	0.1986	0.5561
Arachidonic (C20:4 (5,8,11,14))	22.33 (21.18–23.66)	21.25 (20.33–23.11)	+5%	0.2885	0.5769
Eicosapentanoic–EPA (C20:5 (5,8,11,14,17)).	1.02 (0.75–1.22)	1.12 (0.85–1.22)	-10%	0.9454	0.9672
Docosapentaenoic–DPA C22:5(7,10,13,16,19)	3.60 (3.24–4.00)	3.70 (3.25–4.06)	-3%	0.5365	0.7299
Docosahexaenoic–DHA (C22:6 (4,7,10,13,16,19))	7.71 (6.29–8.50)	7.52 (4.67–9.62)	+2%	0.9672	0.9672

^**$**^ Fatty acids are reported as relative percentages of fatty acid methyl esters (% rel.) obtained by gas chromatographic analysis and calibration procedure, after erythrocyte membrane isolation and work-up as previously reported.^18^ See Methods for the experimental details.

*—Median with IQR

**-Differences between GDM and NGT groups

### Statistical analysis

Continuous variables were presented as medians with the corresponding interquartile ranges (IQR). In order to compare GDM patients and healthy control group, the Mann-Whitney rank test. Benjamini-Hochberg procedure was applied in order to calculate False Discovery Rate (FDR). Linear regression model was performed in order to evaluate the relationship between fatty acids and biochemical parameters. A *p* value lower than 0.05 or FDR lower than 0.15 were considered as statistically significant. STATISTICA 13.0 (StatSoft, Tulsa, OK, USA) was used for statistical analysis.

### Power analysis

The study design enabled detection of a difference between the study and control group no greater than -1 or +1 standard deviation with power 0.8 at type I error probability equaling 0.05.

## Results

The clinical and biochemical characteristics of the groups are given in Table 1. These results indicate that the studied groups did not differ significantly in respect of age, BMI, insulin concentration, HOMA-IR HOMA-ß, QUICKI and LDL levels.

In contrast, OGTT 120’ (160 mg/dL vs 99.6 mg/dL), CRP (3.5 mg/L vs 1.5 mg/L; *p* = 0.0119), total cholesterol (259.9 mg/dL vs 219.5 mg/dL; *p* = 0.0070), and triglyceride (215.9 mg/dL vs 157.6 mg/dL; *p* = 0.03980) levels were significantly higher in the GDM group in comparison to the control group. The concentrations of FPG and HDL were higher in the GDM group however, these differences were at a borderline of significance (85 mg/dL vs 82 mg/dL *p* = 0.0565; 74.1 mg/dL vs 61.4 mg/dL; *p* = 0.0802), respectively.

The results are shown in Table 2. Among the 14 measured fatty acids demonstrating the representative membrane lipidomic cluster, the level of the three SFAs (myristic, palmitic, stearic acids) tends to decrease in GDM patients, with the percentage content of stearic acid changing significantly. The relative content of MUFA tends to increase, except for oleic acid, and the vaccenic acid content is significantly increased in erythrocyte membranes of GDM group in comparison with control group (Table 2). Sapienic acid content was found to be insignificantly higher in the GDM group (+29%).

The Spearman’s correlation analysis between clinical data and fatty acids concentration in erythrocyte membranes in the GDM and NGT group was performed (Table 3). In this analysis the *cis*-vaccenic acid content positively correlated with GDM patients BMI (R = -0.39; *p* = 0.0442), and age (R = 0.56; p = 0.0012) and in NGT group vaccenic acid correlated negatively with FPG (R = -0.7, p = 0.0358), insulin (R = -0.72; p = 0.0298) and HOMA-IR (R = -0.67; p = 0.0499), and positively with QUICKI (R = 0.67; p = 0.0499), while stearic acid correlated negatively with FPG (R = -0.42; *p* = 0.03) and positively with LDL levels (R = 0.38; *p* = 0.04) in GDM group. In NGT group stearic acid correlated positively with OGTT (R = 0.89; p = 0.0188).

**Table 3:** Spearman’s correlation coefficient between clinical data and fatty acids concentration in erythrocyte membranes in the GDM and NGT group.

	Vaccenic acid				Stearic acid				Sapienic acid				Alpha-linolenic acid			
	GDM		Control		GDM		Control		GDM		Control		GDM		Control	
	R	*p*	R	*p*	R	*p*	R	*p*	R	*p*	R	*p*	R	*p*	R	*p*
**Age [years]**	**0.56**	**0.0012**	0.00	1.0000	-0.12	0.5260	-0.36	0.4852	**0.54**	**0.0016**	0.00	1.0000	0.15	0.4260	-0.60	0.2103
BMI* **[kg/m**^**2**^**]**	**0.39**	**0.0442**	0.50	0.3910	-0.17	0.4033	0.30	0.6238	0.21	0.2830	-0.70	0.1881	0.15	0.4471	-0.60	0.2848
**FPG [mg/dL]**	0.11	0.5948	**-0.70**	**0.0358**	**-0.42**	**0.0304**	-0.35	0.3558	0.11	0.5853	-0.42	0.2646	0.14	0.4826	-0.25	0.5165
**OGTT 120’ [mg/dL]**	-0.07	0.7272	-0.14	0.7872	-0.15	0.4721	**0.89**	**0.0188**	0.04	0.8525	-0.60	0.2080	0.13	0.5113	0.26	0.6228
**Insulin [μIU/mL]**	-0.03	0.8836	**-0.72**	**0.0298**	0.05	0.7712	-0.50	0.1705	0.05	0.7794	0.03	0.9322	0.01	0.9416	0.07	0.8647
**HOMA-IR**	0.05	0.8065	**-0.67**	**0.0499**	-0.11	0.5958	-0.48	0.1875	0.17	0.4008	0.20	0.6059	-0.01	0.9542	0.12	0.7650
HOMA-B	-0.07	0.7188	-0.37	0.4685	0.25	0.2045	0.14	0.7872	0.04	0.8418	0.09	0.8717	-0.00	0.9904	0.14	0.7872
**QUICKI**	-0.05	0.8065	**0.67**	**0.0499**	0.11	0.5958	0.48	0.1875	-0.17	0.4008	-0.20	0.6059	0.01	0.9542	-0.12	0.7650
CRP [mg/dL]	-0.29	0.1085	-0.33	0.3807	0.03	0.8683	-0.17	0.6682	-0.10	0.6076	0.05	0.8984	0.22	0.2279	-0.07	0.8647
Total Cholesterol [mg/dL]	-0.02	0.9091	-0.32	0.4064	0.24	0.1967	0.07	0.8647	-0.14	0.4661	0.18	0.6368	0.08	0.6726	-0.17	0.6682
HDL [mg/dL]	0.00	1.0000	-0.22	0.5755	-0.23	0.2215	*-0*.*65*	*0*.*0581*	-0.04	0.8438	0.42	0.2646	0.14	0.4687	0.05	0.8984
**LDL [mg/dL]**	-0.09	0.6113	-0.23	0.5457	**0.38**	**0.0364**	-0.02	0.9661	-0.22	0.2274	0.12	0.7650	-0.06	0.7678	-0.08	0.8312
**TG [mg/dL]**	0.24	0.2030	**-0.73**	**0.0246**	0.05	0.7712	0.30	0.4328	0.18	0.3419	-0.13	0.7324	0.14	0.4594	-0.37	0.3317

*-Assessed before pregnancy

BMI—body mass index; HbA1C —glycated hemoglobin; HOMA-IR—homeostasis model assessment of insulin resistance; HOMA- β homeostasis model of assessment of beta cell function.

In the multiple linear regression model (Table 4), after adjusting to putative cofounders (age, BMI, CRP, OGTT results and lipid profile), levels of vaccenic acid and alpha–linolenic acid were significantly higher in GDM group versus control one. Additionally, BMI and glucose concentration in 120'-OGTT were positively associated with levels of *cis*-vaccenic acid and alpha–linolenic acid. The latter one was also positively associated with levels of HDL in multiple regression analysis.

**Table 4:** Multiple linear regression coefficient between clinical data and fatty acids concentration in erythrocyte membranes in the GDM and NGT.

	Vaccenic acid		Stearic acid		Sapienic acid		Alpha-linolenic acid	
	Beta	p	Beta	p	Beta	p	Beta	p
**GDM vs control**	**0.71**	**0.0312**	-0.20	0.5976	-0.06	0.8688	**0.87**	**0.0045**
**Age [years]**	0.17	0.2948	-0.14	0.5026	0.34	0.1066	0.23	0.1248
**BMI [kg/m**^**2**^**]**	**0.93**	**0.0003**	-0.30	0.2709	-0.01	0.9638	**0.93**	**0.0001**
**CRP [mg/dL]**	-0.33	0.1008	0.24	0.3168	-0.05	0.8454	*-0*.*33*	*0*.*0664*
**FPG [g/dL]**	-0.30	0.1759	-0.16	0.5491	0.39	0.1563	*-0*.*33*	*0*.*0993*
**Total Cholesterol [mg/dL]**	**0.78**	**0.0229**	-0.47	0.2394	0.05	0.9068	**0.98**	**0.0023**
**HDL [mg/dL]**	0.28	0.1274	-0.19	0.3866	0.03	0.8748	**0.34**	**0.0455**
**LDL [mg/dL]**	-0.23	0.3187	0.13	0.6524	0.01	0.9855	-0.24	0.2395
**TG [mg/dL]**	-0.24	0.1945	0.19	0.4021	0.05	0.8309	-0.22	0.1904

## Discussion

The metabolic and dietary linkages between SFA and MUFA are very important from a pathophysiological point of view. These fatty acids are involved in membrane organization and properties such as membrane fluidity and permeability, being the cell membrane functionality highly dependent on a balance between quantities and qualities of fatty acids [24, 25].

**Fig 1:**
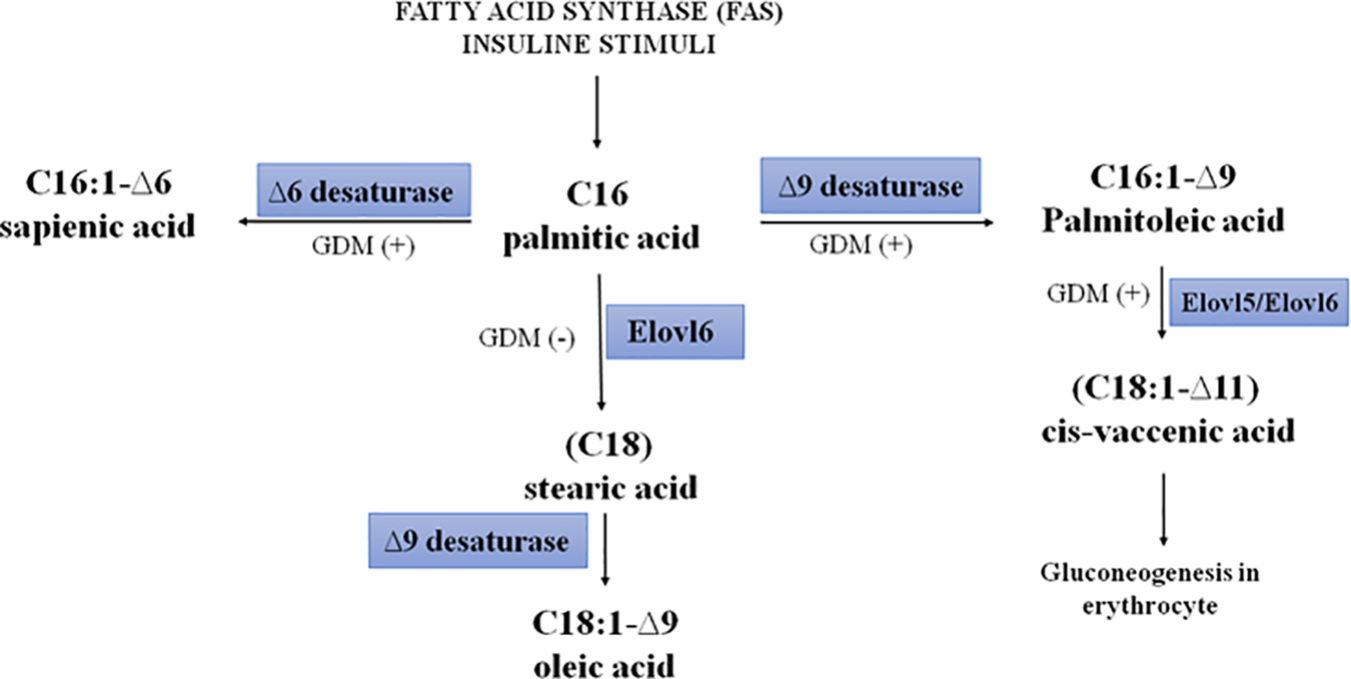
Possible influence of GDM on pathway of metabolic transformation of palmitic acid to *cis*-vaccenic, sapienic and oleic acids [30].

The SFA and MUFA families are the most involved in metabolic deregulation and are connected with insulin resistance and related diseases [26, 27]. According to Mozaffarian *et al*, both SFAs and MUFAs can affect the metabolic pathways related to diabetes [28]. Our present findings indicate that the level of vaccenic acid in erythrocyte membranes increased significantly by about 14% in women diagnosed with GDM. This is the first time that such an association between impaired *cis*-vaccenic acid content in the erythrocyte membrane and GDM development has been evidenced. It is important to note that 11- *cis* octadecenoic acid, also known as *cis*-vaccenic acid (VA), is synthesized in humans from palmitic acid (hexadecanoic acid) which is converted to palmitoleic acid (9-*cis* hexadecenoic acid) by stearoyl-CoA-desaturase (Δ9-desaturase) (SCD; EC 1.14.19.1) and then to vaccenic acid by elongase (ELOVL5/6 EC 6.2.1.3). Fig 1 presents this metabolic transformation, starting from palmitic acid and hypothetic pathway of metabolic transformation of palmitic acid to *cis*-vaccenic, sapienic and oleic acids in GDM. In contrast, the 11-*trans* vaccenic acid it is widely present in ruminants and is acquired by humans in abundance from consumed dairy products [29]. In our studies, the 11-*cis* VA was the only isomer detected in erythrocyte membranes.

It was reported recently that a higher concentration of *cis*-VA in plasma was associated with a reduced risk of T2DM, lower fasting glucose and better insulin sensitivity in male Sprague–Dawley rat models [31], and in T2DM middle-aged and elderly Chinese patients [32]. Chen *et al* studied circulating (serum) fatty acids and detected impaired composition of fatty acids not only in women with GDM, but also in women with less severe glucose intolerance. A strong correlation between severity of maternal hyperglycemia and concentration of individual serum fatty acids in the third trimester was demonstrated however, plasma lipids are more dependent on dietary intakes than found in tissues or cell membranes [33].

In the reported study, women with GDM demonstrated higher lipids concentration (TG, LDL, HDL and total cholesterol) than the control group (see Table 1). The fatty acid cluster composition (expressed as single fatty acid percentage over the 14 fatty acids of the cluster) revealed important metabolic differences in the profiles of women with GDM. The increased level of VA in our study also corresponds to an unchanged level of fasting glucose between GDM and NGT group (Table 3).

In our opinion, the observed increase of *cis*-VA content is a sign of increased metabolic transformation of palmitic acid through the stearoyl-CoA-desaturase (Δ9-desaturase) and elongase activities in GDM women (see Fig 1), and the *cis-*VA can be transferred to the fetus, thus predisposing it to the enzymatic induction of the biosynthesis of palmitoleic acid, which is already marked as risk a factor for metabolic abnormalities and new onset of diabetes [28]. As reported earlier, a higher level of palmitoleic acid has beneficial effect on insulin sensitivity in case of metabolic diseases, and its administration in animal models being effective for reducing insulin resistance and hepatic lipid accumulation [34]. In GDM, the increase in *cis-*VA can be interpreted as the metabolic transformation of palmitoleic acid, and consequent loss of its beneficial effects. Therefore, the *cis*-VA has a biomarker role in GDM women, indicating the metabolic cascade that is activated in these subjects.

There are also known other non-lipids compounds which could play the role of biomarkers in GDM. One of these is galanin studied by Zhang et. al [35]. Galanin was analyzed by an enzyme-linked immunosorbent assay. The plasma galanin level was higher in GDM patients compared with NGT. What more, authors demonstrated the significant positive correlation between galanin and FPG, 1-h glucose, BMI in GDM women and the significant positive correlation between galanin and BMI in NGT one. According, to the authors the higher level of galanin demonstrated in GDM could be responsible for an adaptation to the rise of glucose and weight associated with GDM. Furthermore, the increased level of galanin is connected with higher BMI before gestation [36]. In turn, Georgiou *et al*. [37] investigated several biomarkers including endocrine and metabolic hormones, cytokines and chemokines and markers of oxidative stress at the beginning of pregnancy (at 11 weeks of gestation) and at 28 weeks of gestation. They observed elevated levels of plasma insulin and reduced plasma adiponectin concentration compared to controls [37]. In comparison, our study on erythrocyte membrane fatty acids state can be more reflective, because the red blood cell membrane composition reflects the 2–3 months dietary intake.

An important observation in our GDM cohort is that these women were not obese (taking BMI before pregnancy into account), which is noteworthy because, in general, the activation of Δ9-desaturase, responsible for increased palmitoleic acid levels, is connected to the overweight/obesity status [28]. Moreover, in earlier studies of overweight GDM patients the changes of their fatty acid profiles were different, and connected with the obesity traits [13].

Pregnant women are susceptible to systemic inflammation, metabolic disorders, and oxidative stress, which can, in turn, lead to complications in maternal and fetal life [38]. In our study on GDM patients in comparison to NGT, increased CRP level was observed. These data may suggest that in women with gestational diabetes, the CRP concentration is primarily related to the degree of adiposity until the second trimester and that thereafter impaired glucose metabolism appears to be the predominant predictor of changes in CRP [39]. Our findings indicate that the stearic acid level in GDM women was decreased in comparison to the NGT group. This adds further support to the hypothesis of the metabolic shift in GDM subjects. In fact, the faster conversion of palmitic acid to *cis*-VA results in the reduced transformation of palmitic acid into stearic acid (elongation step), as shown in Fig 1. Summarizing, in GDM subjects, the metabolism of palmitic acid is more directed towards the palmitoleic-vaccenic acid pathway than to the stearic-oleic acid pathway.

A particularly important observation concerns changes of sapienic acid, which was found as high as 29% in the GDM group than NGT subjects, although without statistical significance. Sapienic acid is a metabolite of palmitic acid produced by Δ6-desaturase and was observed for the first time in erythrocyte membranes by Ferreri *et al* [18]. The effect of sapienic acid in GDM pathogenesis has not been studied so far, therefore, this research area looks promising and requires further study. Relatively widespread results were observed among the studied GDM patients, with the crude results differing considerably from very low concentration to very high.

The analysis of multiple regression (Table 4), after adjusting to corresponding cofounders including age, BMI, CRP, OGTT and lipid profile, revealed the significantly higher presence of two fatty acids, namely cis-vaccenic and α-linolenic acid. These results confirm our findings of cis-VA function in remodeling concomitant with GDM. This also supports our hypothesis on changing of metabolism palmitic to palmitoleic acid (Fig 1) [21,32]. The α-linolenic acid increase in our study is in line with other reports on its function in metabolic diseases [33].

The correlations presented in Table 3 confirm that: i) the increase of vaccenic acid is due to the metabolic pathway of palmitic acid transformation and is positively correlated to BMI, since an increase of BMI can indicate *cis*-vaccenic acid formation from palmitoleic acid, which is consequently increased; ii) the decrease of stearic acid is positively correlated to LDL levels, which indicates lipoprotein formation, whereas it is negatively correlated with FPG, thus indicating that the metabolic shift of stearic acid diminution has a role in the glucose management.

Overall, our results show SFA-MUFA families are more involved in metabolic diseases like GDM; in fact, no significant changes of PUFA levels were found. In GDM, it seems that a crucial metabolic shift occurs in the biotransformation of palmitic acid between the two pathways depicted in Fig 1. The study requires validation due to its pilot / exploratory nature.

In our GDM cohort, an interesting lipidomic profile was depicted with stearic and *cis*-vaccenic acids as dual biomarkers of a specific SFA-MUFA conversion based on the coupling of Δ9-desaturase and elongase enzymes. This profile has to be confirmed with a larger number of patients, and further studies will open new perspectives for the control of *cis*-VA and palmitoleic acid levels in GDM. Indeed, the effects of a proper diet or early PUFA supplementation during pregnancy in case of GDM can be corroborated with the results at the molecular level using the lipidomic profile.

The strength of this preliminary study comes from the fact that, for the first time, the lipids profile of erythrocyte membranes in pregnant women has been explored and rationalized. The differences in fatty acids content between the lipidomic membrane profiles in erythrocyte membranes of GDM women, and normoglycemic pregnant patients have been analyzed in terms of potential mechanistic pathways responsible for the established correlation. The increased content of cis-VA is of special interest since this isomer of VA can only be the result of increased synthesis in the case of GDM in comparison to normoglycemic pregnancy (Fig 1), and its delivery with food and/or supplementation can be excluded.

The next important element of this report regards the size of the study group. Here, we recruited well- defined homogenous group of pregnant women in terms of biochemical and metabolomics status, and divided them, according to the OGGT test, into GDM and controls (normoglycemic pregnant patients).

A relatively small patients group involved into the study, although it is accepted from the point of view of statistical evaluation, can be considered a major limitation of this report. However, in order to reduce possibility of false positive or negative correlations, we applied a strict FDR approach for the analysis, followed by the multiple linear regression analysis thus explaining limited factors connected with fatty acids profile in erythrocytes membrane. One can also assume the next limitation of the presented study in the fact that fatty acid profiles were measured only at one point (24–28 week) during pregnancy and not followed further. To address this, our further study is designed in such a way as to examine the lipid profiles of pregnant women in 3 time points of pregnancy and follow up to explore the dynamics of remodeling of membranes during and after pregnancy.

In conclusion, this study documented for the first time an abnormal profile of erythrocyte membranes fatty acids in GDM women diagnosed according to a standard OGTT test, revealing a statistically significant changes in cis-VA and stearic acids content. This knowledge can influence on both the mother and her offspring diagnosis and treatment.

## Abbreviations:

BMI: body mass index
CRP: C-reactive protein
FPG: fasting plasma glucose
FAME: fatty acids methyl esters
GC: gas chromatography
GDM: gestational diabetes mellitus
HbA1C: glycated hemoglobin
HOMA-IR: homeostasis model assessment of insulin resistance
HOMA- β: homeostasis model assessment of β cell function
HDL: high density lipoprotein
IQR: interquartile range
LDL: low density lipoprotein
MUFA: monounsaturated fatty acids
Na_2_EDTA: Disodium ethylenediaminetetraacetate dehydrate
NGT: normal glucose tolerance
PUFA: polyunsaturated fatty acids
SFA: saturated fatty acids
T2DM: type 2 diabetes mellitus
TG: triglycerides
VA: vaccenic acid
WHO: World Health Organization
